# A margin-based analysis of the dosimetric impact of motion on step-and-shoot IMRT lung plans

**DOI:** 10.1186/1748-717X-9-46

**Published:** 2014-02-05

**Authors:** Benjamin J Waghorn, Amish P Shah, Justin M Rineer, Katja M Langen, Sanford L Meeks

**Affiliations:** 1 Department of Radiation Oncology, UF Health Cancer Center at Orlando Health, 1400 South Orange Avenue MP 730, Orlando, Florida 32806, USA

**Keywords:** IMRT, Motion, Step-and-shoot, Lung, Dosimetry, 87.55.dk

## Abstract

**Purpose:**

Intrafraction motion during step-and-shoot (SNS) IMRT is known to affect the target dosimetry by a combination of dose blurring and interplay effects. These effects are typically managed by adding a margin around the target. A quantitative analysis was performed, assessing the relationship between target motion, margin size, and target dosimetry with the goal of introducing new margin recipes.

**Methods:**

A computational algorithm was used to calculate 1,174 motion-encoded dose distributions and DVHs within the patient’s CT dataset. Sinusoidal motion tracks were used simulating intrafraction motion for nine lung tumor patients, each with multiple margin sizes.

**Results:**

D_95%_ decreased by less than 3% when the maximum target displacement beyond the margin experienced motion less than 5 mm in the superior-inferior direction and 15 mm in the anterior-posterior direction. For target displacements greater than this, D_95%_ decreased rapidly.

**Conclusions:**

Targets moving in excess of 5 mm outside the margin can cause significant changes to the target. D_95%_ decreased by up to 20% with target motion 10 mm outside the margin, with underdosing primarily limited to the target periphery. Multi-fractionated treatments were found to exacerbate target under-coverage. Margins several millimeters smaller than the maximum target displacement provided acceptable motion protection, while also allowing for reduced normal tissue morbidity.

## Introduction

During radiation therapy, intrafraction motion has the potential to affect target dosimetry [1], sometimes with significant consequences to target dose coverage [2,3]. This is especially true during intensity modulated radiation therapy (IMRT) [4], where increases in target conformality can produce more desirable dose distributions for the static patient, but at the same time can make the target more susceptible to underdose due to intrafraction target motion [5]. A number of techniques exist to minimize the effects of intrafraction motion, with the addition of a planning target volume (PTV) being the most commonly used technique to maintain uniform dosimetry to the target even during motion [6].

Several methods have been developed to estimate the dosimetric impact of intrafraction motion including Monte Carlo simulations [7], experimental phantom measurements [8,9] and computational methods [10-12]. One recent computational technique applied a given motion track to a static treatment plan to estimate the three-dimensional (3D) dose distribution within the patient anatomy, allowing for performance of DVH-based analyses [10]. This current study utilizes the same technique to investigate the impact of intrafraction motion on SNS IMRT, specifically to study the relationship between motion and dosimetric effect. Motion and planning parameters were varied to test a range of combinations, some of which were similar to those used clinically while others were, by design, beyond those used clinically. The CT dataset and target contours from nine lung cancer patients were investigated, encompassing a representative range of tumor shapes and sizes. In total 1,174 motion-encoded dose distributions were calculated for different sinusoidal motion track, margin size, and treatment plan combinations for the nine patients. The cumulative effects of motion during multi-fractionated deliveries were also considered.

## Methods and materials

Under an Institutional Review Board (IRB)-approved protocol, nine lung cancer cases were retrospectively reviewed for this motion study, all of which had been selected for 6 MV SNS IMRT treatments.

### Treatment planning

The initial target volume determination for the nine patients were contoured by a radiation oncologist using information from the individual 4DCT phases, the average CT, and the maximum intensity projection (MIP) reconstruction from the 4DCT, resulting in volumes ranging from 22.2 to 503.1 cm^3^ (average volume ± 1 standard deviation = 228.9 ± 185.4 cm^3^). Target and PTV combinations with target to PTV margin expansions of 0, 3, 6, 10 and 15 mm were uniformly applied. Clinically relevant optimization objectives were used to create SNS IMRT treatment plans for each PTV/target combination within a research version of a commercial treatment planning system (Pinnacle, Version 8.1x, Philips Healthcare, Andover, MA). The treatment plans were calculated on the patient’s free-breathing CT. The number of beams (all coplanar), treatment prescription, average number of segments per beam, and the average patient plan MU/dose (assumed to be proportional to the plan complexity [13]) are summarized in Table 1.

**Table 1:** Patient prescription and treatment plan information

**Patient number**	**Prescription dose (cGy)**	**Number of fractions**	**Number of beams**	**Primary target volume (cm**^**3**^**)**	**Margin sizes (mm)**	**Average segments per beam**	**Average MU/dose (MU/cGy)**
1	7,000	35	8	22.2	0,3,6,10 & 15	4.0	2.31
2	7,000	35	6	25.9	0,3,6,10 & 15	3.9	2.06
3	4,500	30	6	476.5	0,3,6 & 10	7.8	3.37
4	7,000	35	9	503.1	0,3,6 & 10	9.2	3.33
5	7,400	37	6	325.4	0,3,6 & 10	10.0	2.29
6	7,000	35	6	238.5	0,3,6 & 10	11.5	2.54
7	6,000	30	8	99.2	0,3,6 & 10	8.3	2.56
8	5,000	20	5	74.4	0,3,6 & 10	9.6	1.70
9	7,400	37	11	294.9	0,3,6 & 10	6.4	3.29

### Calculating the motion-encoded dose distribution

A computational algorithm was developed in MATLAB to estimate the dosimetric effect of intrafraction motion [10]. Based upon a chosen motion track, individual segment fluence maps were shifted in the opposite direction of the physical target’s beams-eye-view displacement in order to account for this motion during each delivered monitor unit (MU). MU timing was calculated using a 400 MU/minute dose rate, assuming a 1 second interval between each segment (a typical inter-segment treatment time acquired from DynaLog files) and a 40 second interval between each gantry angle. A final dose calculation was performed within the CT dataset using the modified fluence maps, creating the 3D motion-encoded dose distribution. Previous work has validated the accuracy of the motion-encoded dose distributions [10].

### Motion tracks

Ideally, this analysis would be performed using clinically acquired tumor motion tracks. It has been demonstrated, however, that respiratory motion can be approximated as sinusoidal motion of the form shown in Eq. 1 [14]:

(1) $$\begin{array}{ll} \mathrm{Displacement}\left(t\right)\left[\mathrm{mm}\right] & =A\left[\mathrm{mm}\right]\cdot \sin\left(\frac{2\pi }{T\left[\sec\right]}t\left[\sec\right]+\varphi \left[{}^{\circ}\right]\right)\\ & +\left(D\left[\mathrm{mm}/\sec\right]\cdot t\left[\sec\right]\right)+O\left[\mathrm{mm}\right] \end{array}$$

Each parameter from Eq. 1 (namely amplitude (A, half peak-to-peak motion), drift (D), offset (O), period (T) and phase (φ)) was investigated separately to determine its effect on the dosimetry. The specific motion variables for each patient are shown in Table 2, with default values of A = 7.2 mm, T = 3.8 sec [14], φ = 0°, D = 0 mm/min and O = 0 mm being used when not being explicitly stated. For example, when the effect of target drift was being investigated, values of A = 7.2 mm, T = 3.8 sec, φ = 0° and O = 0 mm were held constant while the drift rate was varied as shown in Table 2, column 4. For each motion track, data was calculated for every margin size listed in Table 1, creating a total of 1,174 motion-encoded dose distributions.

**Table 2:** Motion track parameters per patient

**Patient number**	**Motion direction**	**Amplitude (half peak-to-peak, mm)**	**Drift (mm/min)**	**Offset (mm)**	**Period (sec)**	**Phase (radians)**
1	SI/AP/3D	0, 3, 5, 7.2, 10, 15	-3, -1.6, 0, 1.6, 3	-3, -2, -1, 0, 1, 2, 3	2.2, 3.8, 6.4, 10	0, 2π/5, 4π/5, 6π/5, 8π/5
2	SI/AP/3D	0, 3, 5, 7.2, 10, 15	-3, -1.6, 0, 1.6, 3	-3, -2, -1, 0, 1, 2, 3	2.2, 3.8, 6.4, 10	0, 2π/5, 4π/5, 6π/5, 8π/5
3	SI/AP	0, 5, 7.2, 10, 15	-2, 0, 2	0, 3, 5	3.8	0
4	SI/AP	0, 5, 7.2, 10, 15	-2, 0, 2	0, 3, 5	3.8	0
5	SI/AP	0, 5, 7.2, 10, 15	-2, 0, 2	0, 3, 5	3.8	0
6	SI/AP	0, 5, 7.2, 10, 15	-2, 0, 2	0, 3, 5	3.8	0
7	SI/AP	0, 5, 7.2, 10, 15	-2, 0, 2	0, 3, 5	3.8	0
8	SI/AP	0, 5, 7.2, 10, 15	-2, 0, 2	0, 3, 5	3.8	0
9	SI/AP	0, 5, 7.2, 10, 15	-2, 0, 2	0, 3, 5	3.8	0

### Intrafraction motion dosimetric impact analysis

Motion-encoded target DVHs were calculated from the motion-encoded dose distribution, and were compared to the static DVHs by calculating target ∆D_95%_ and ∆D_05%_ values; these represent the difference between static and motion-encoded D_95%_ and D_05%,_ respectively. By definition, ∆D_95%_ and ∆D_05%_ equal unity if the motion had no dosimetric impact.

**Figure 1:**
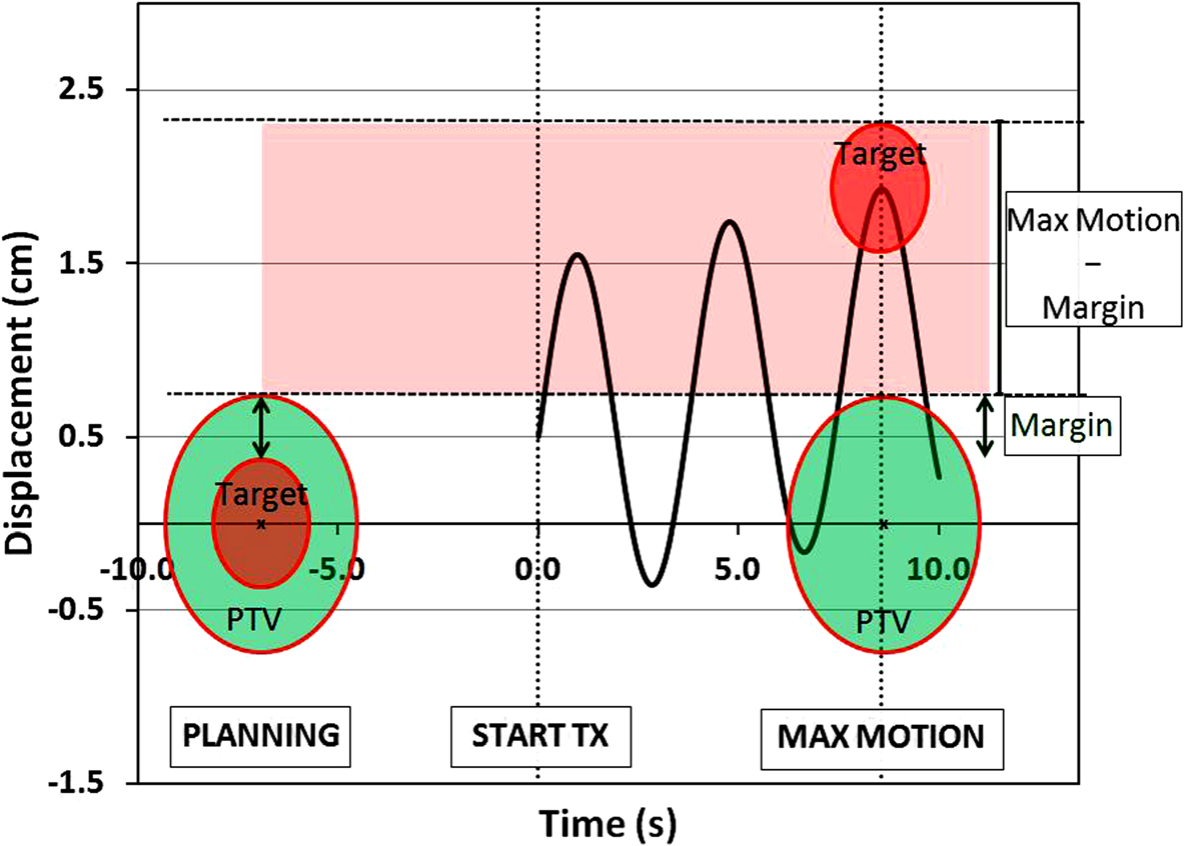
A schematic representation of intrafraction target motion.

The quantity 'Max Displacement – Margin’ (mm) was calculated for each motion calculation and represents the maximum displacement of the target outside the PTV (Figure 1). This parameter was used to assess the effectiveness of the margin concept for motion management, with an acceptable target deviation selected as ∆D_95%_ > 0.97 (i.e. less than a 3% reduction in D_95%_). The study was designed to provide a wide range of sinusoidal tumor displacements, encompassing a majority of the maximum tumor displacement expected clinically. Therefore, analysis of the 'Max Displacement – Margin’ parameter was used to create a new approach to the margin size decision making process.

### Multi-fractionation analysis

To determine the cumulative impact of motion on multiple fractions, accumulated DVHs were calculated for the following scenarios:

1) Patient 2 (0 mm margin), five fractions each with the same target motion track (15 mm amplitude SI), but with different starting phases.

2) Patient 5 (0 mm margin), with seven randomly selected SI motion tracks.

3) Patient 2 (0 mm margin), with thirty randomly selected SI motion tracks.

 Individual fraction motion-encoded dose distributions and DVHs were calculated, as well as the accumulated dose distribution and DVH.

## Results

The static plan complexity (characterized by the number of MUs required to deliver a cGy of dose) [13] increased approximately linearly with increasing PTV volume (Table 1), with the linear least square best fit function shown in Eq. 2 (r^2^ = 0.66). Larger PTVs required more complex static treatment plans (Table 1).

(2) $$\begin{array}{ll} \mathrm{MU}/\mathrm{Dose}\left(\mathrm{MU}/\mathrm{cGy}\right)= & 1\times 10^{‒3}\\ & \times \mathrm{PTV}\;\mathrm{Volume}\;\left({\mathrm{cm}}^{3}\right)\\ & +1.9 \end{array}$$

**Figure 2:**
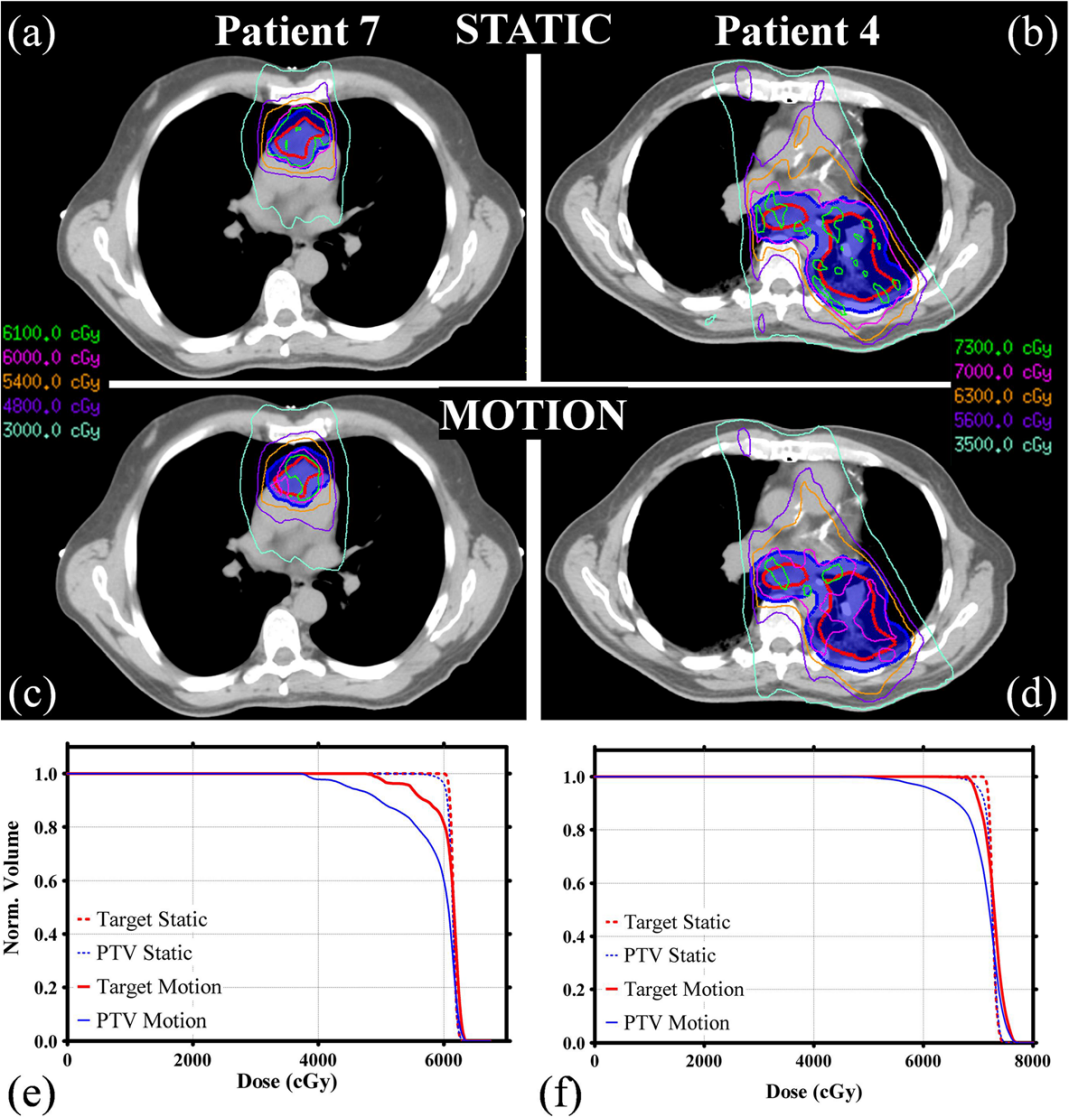
**Dosimetric comparison between patient 7 and patient 4.** Sample static treatment plans for patients 7 (6 mm margin) and 4 (10 mm margin) are shown in **a** and **b**, respectively. The dosimetric effect of a 15 mm amplitude SI sinusoidal motion track is shown in **c** and **d** for patient 7 and 4, respectively. The corresponding target (thick lines) and PTV (thin lines) DVHs for these two cases are shown in **e** and **f**, both without motion (dashed lines) and with motion (solid lines).

Two example treatment plans are shown in Figures 2a and 2b, along with 15 mm amplitude SI motion-encoded dose distributions in Figures 2c and 2d, respectively. The corresponding target and PTV DVHs for these two cases are shown in Figures 2e and 2f.

Motion amplitudes (half peak-to-peak displacement) used in this study ranged from 0 to 15 mm (Table 2). These motion tracks corresponded to 'Max Displacement – Margin’ values ranging from -15 mm (15 mm margin, 0 mm amplitude) to +15 mm (0 mm margin, 15 mm amplitude). Similarly, drift velocities from -3 to +3 mm/min were investigated with the absolute target displacement dependent on the treatment time. The average target displacement due to drift was 9.6 ± 4.8 mm ('Max Displacement – Margin’ values ranged from -7.8 to +24.8 mm for the default 7.2 mm amplitude drifting tracks). Offsets of -3 to +5 mm were also studied ('Max Displacement – Margin’ ranged from -7.8 to +12.2 mm with 7.2 mm amplitude).

**Figure 3:**
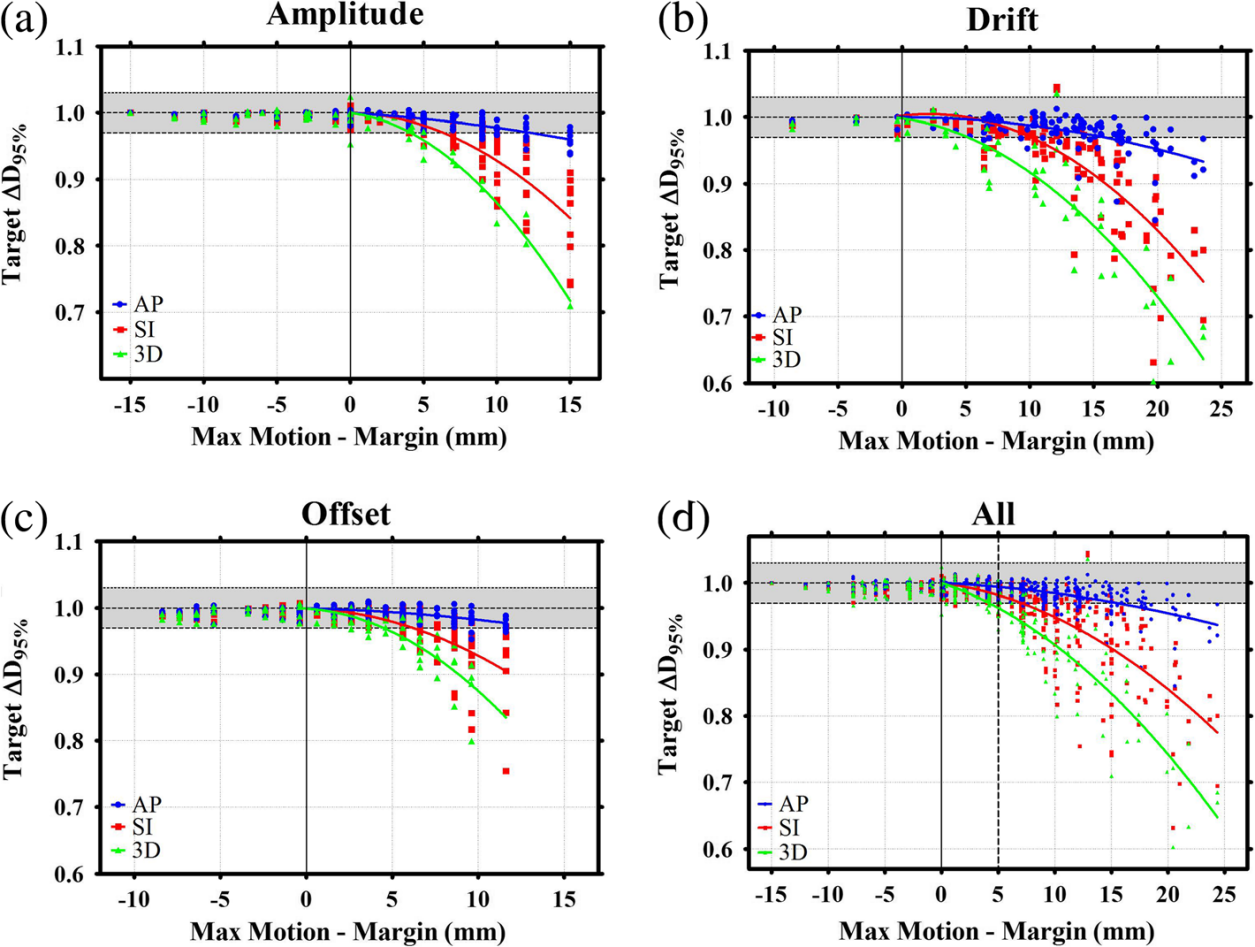
**The effect of varying the motion amplitude, drift and offset on ∆D**_**95% **_**are shown separately in a, b and c respectively, and combined in d.** Each of the plots shows the effect of increasing the target displacement outside the PTV ('Max Displacement – Margin’) on target D_95%_. The shaded regions represent < ±3% change in D_95%_. Least-square quadratic fits are shown for each motion direction.

Figure 3 shows the effect of 'Max Displacement – Margin’ on target D_95%_, with the results from the amplitude, drift and offset studies shown separately in Figures 3a, 3b and 3c respectively, and combined in 3d. Least-square fits of a quadratic function were applied to the data for each test and motion direction (for 'Max Displacement – Margin’ values > 0), with the resultant curves shown. All of the data are included in Figure 3d, ranging from the motion track with 15 mm margin and 0 mm amplitude motion to the worst-case scenario of zero margin and 3 mm/min drift. By converting these data points to 'Max Displacement – Margin’ the full spectrum of motion tracks studied here can be compared together without skewing the results; these outliers simply form the extremes within the plot, and help to define the relationship between displacement, margin size and target dosimetry.

It can be seen from Figure 3 that the dosimetric effect of motion is dependent on the direction of motion. For example, when 'Max Displacement – Margin’ was between 14 and 16 mm (14.9 ± 0.4 mm), average ∆D_95%_ values for motion in the AP, SI and 3D directions were 0.97 ± 0.02, 0.90 ± 0.07 and 0.82 ± 0.8 respectively. Using the least-square best-fit quadratic curves, D_95%_ was reduced by more than 3% (∆D_95%_ < 0.97) when 'Max Displacement – Margin’ exceeded 15.4, 7.0 and 4.1 mm for motion in the AP, SI and 3D directions respectively. The shaded regions in Figure 3 represent a < ±3% change in D_95%_ due to motion. This data can be directly used as a new margin recipe, dependent on the direction and magnitude of the maximum expected tumor displacement.

Changes in target ∆D_95%_ were observed for Patients 1 and 2 with varying periods. ∆D_95%_ values for each individual period deviated from the average by only -3.4% to +1.3%. Similarly, all of the different starting-phase ∆D_95%_ values for each margin size and motion direction were within ±1.4% of the average ∆D_95%_ value.

The effect of motion on D_05%_ was also studied, with only 3.2% of the tracks experiencing more than a 5% increase in ∆D_05%_, and less than 1% increasing by more than 10%. A majority of the high ∆D_05%_ values were caused by 3D motion with AP motion causing the smallest changes. Unlike the results for ∆D_95%_, ∆D_05%_ was relatively independent of the amount of motion present when the motion size was larger than the margin size.

**Figure 4:**
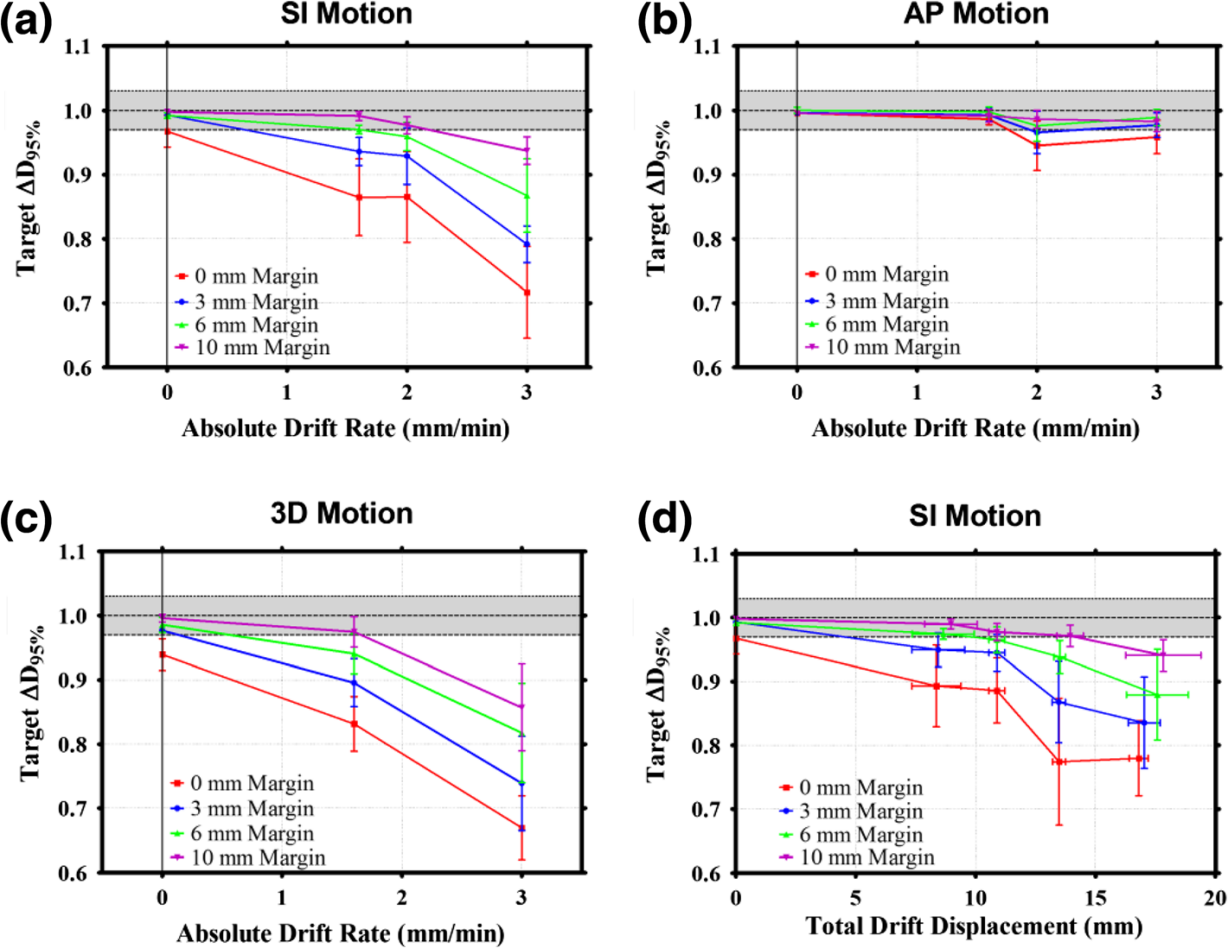
**The effect of varying the drift rate on ∆D**_**95% **_**for SI, AP and 3D motions are shown in a, b and c respectively, as well as the effect of increasing the total SI drift during treatment (d).** Each of the plots shows the effect of changing the drift rate of a 7.2 mm amplitude sinusoidal motion track on target dosimetry for various margin sizes. The shaded regions represent < ±3% change in D_95%_. Note that the total tumor displacement is equal to the drift displacement plus the sinusoidal amplitude.

Figure 4 displays the effect of varying the drift rate (absolute) on target ∆D_95%_ for various margin sizes in the SI, AP and 3D directions (Figure 4a, 4b and 4c, respectively), as well as the effect of increasing the total SI drift during treatment (Figure 4d). In contrast to Figure 3, the data shown in Figure 4 distinguishes directly between margin size and the drift, as opposed to combining the variables to form the 'Max Displacement – Margin’ quantity. With an amplitude of 7.2 mm present in each motion track, almost identical motion direction and margin size dependencies on the target dosimetry are observed, as were seen in Figure 3 and as described above. Figure 4d also takes into account the total treatment time by converting the dose rate into the maximum drift displacement (drift rate × treatment time) for each data point. The maximum total displacement would be the summation of the total drift and 7.2 mm for the sinusoidal amplitude. Similar conclusions can be drawn regarding the effect of margin size on target dosimetry, as described above.

**Figure 5:**
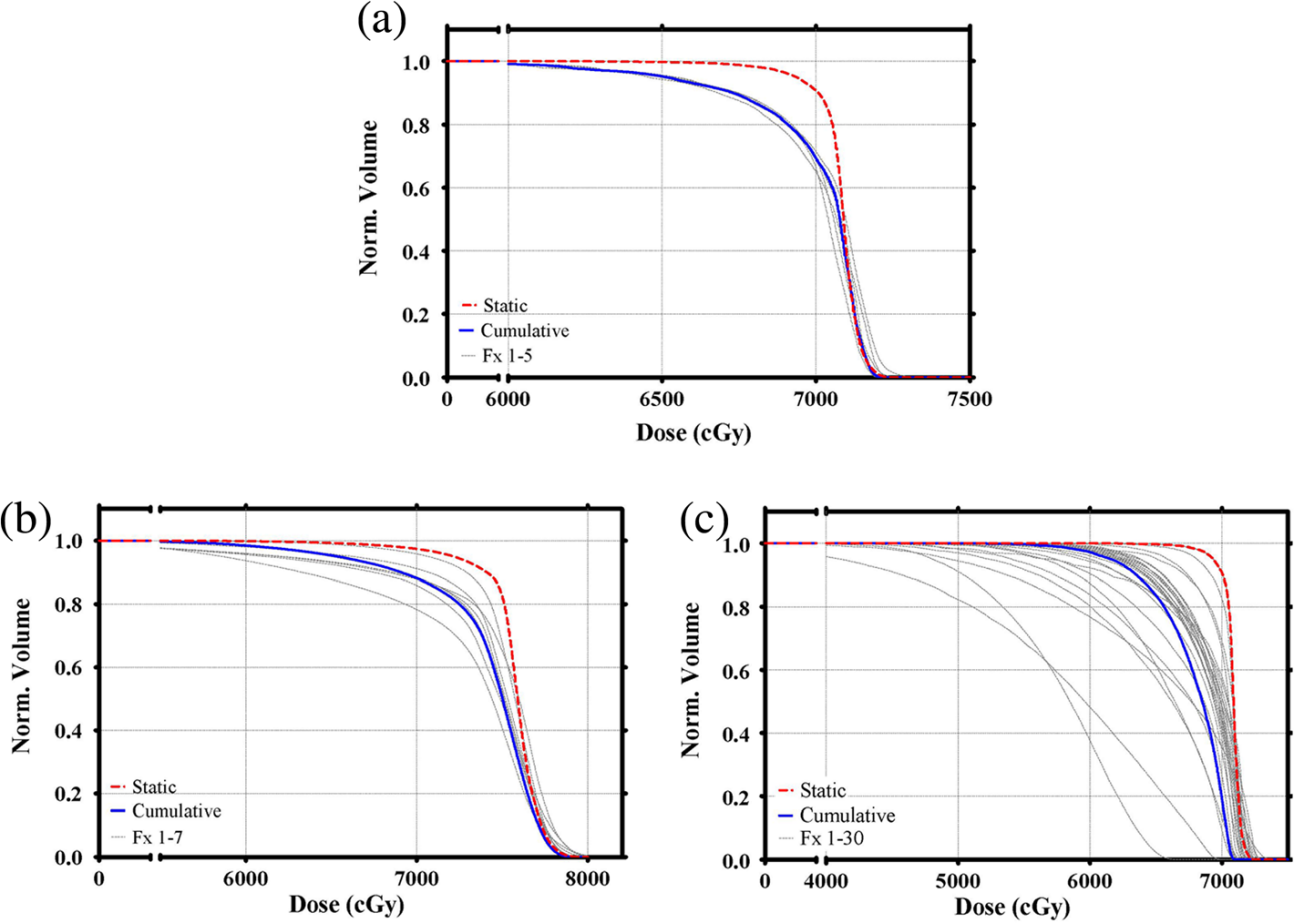
**The dosimetric effects of multi-fractionated treatments are shown with DVHs for the static plan (thick dashed line), the individual fractions (thin lines) and the cumulative dose (thick solid line). a** shows five fractions with the same motion track (15 mm amplitude SI motion), but different starting phases per fraction. **b** and **c** show 7 and 30 fractions respectively, with each fraction experiencing a different, randomly selected, SI motion track.

Finally, the results of the multi-fraction study are shown in Figure 5. Results from the three different scenarios 1, 2, and 3 listed above are shown in Figures 5a, 5b, and 5c, respectively. While the cumulative D_95%_ for multiple fractions was approximately equal to the average D_95%_ of the individual fractions, the cumulative D_05%_ was less than the average D_05%_ of the individual fractions.

## Discussion

This investigation was devised to provide a more thorough understanding of the potentially detrimental dosimetric effects of intrafraction motion and to investigate the effectiveness of target margins to minimize these dosimetric effects. Adequate CTV to PTV margins for treatment of moving lung targets has been extensively discussed in the literature. There are two main thought processes for margin calculations; (1) include all possible target positions during the breathing cycle [15] or (2) make a probabilistic margin calculation based on the target motion [16]. This study presents a third approach as a compromise between the two common techniques, as well as provides a simple method for margin calculation. A range of motion amplitudes and target margins was investigated, incorporating situations where the target remained within the PTV during treatment to where the target deviated up to 24.8 mm outside the PTV. This range was chosen to incorporate the broad spectrum of tumor motions; for example, although an upper lobe tumor may move considerably less than a lower lobe tumor, the smaller displacement data points from this study can be used for analysis, and vice versa. A relatively large upper limit of 24.8 mm displacement is useful to provide a full range of clinically possible motion tracks, although a tumor with this magnitude of displacement would most likely use alternative motion management techniques. In general, changes in D_95%_ were less than 3% if the margin sizes followed Eq. 3.

(3) $$\mathrm{Margin}\;\mathrm{Size}\left(\mathrm{mm}\right)\geq \mathrm{Max}\;\mathrm{Displacement}\left(\mathrm{mm}\right)‒5\;\mathrm{mm}$$

Based on this investigation, the target can be displaced by up to 5 mm outside the original PTV with less than 3% change in the target D_95%_. Additionally, this suggests that there is little benefit in adding a margin equal to or greater than the anticipated maximum displacement, versus a margin that is 5 mm smaller than the extent of motion. Further, this could potentially improve margin-size optimization, allowing for better sparing of normal tissue. Clinically, margins are less likely to be created isotropically as presented in this study, but typically might be larger in the craniocaudal plane than the axial plane, for example. The data presented within this paper is still valid for anisotropic expansions: the formula presented in Equation 3 will need to be separated into SI and AP motion directions to create optimal margin sizes dependent on the three-dimensional nature of the motion.

Equation 3 is not universally true for all motion tracks and for all patients; therefore, care has been taken to study a wide range of different lung tumors in order to make these conclusions as robust as possible. Additional studies considering actual patient motion tracks are warranted to test the integrity of Eq. 3, but are beyond the scope of this current investigation. Equation 3 provides a useful guideline for the treatment planning phase of SNS IMRT under the assumptions that the motion at time of simulation is the same as the motion at time of treatment.

In Figure 3 it can be seen that the detrimental effects of motion are strongly dependent on motion direction. AP motion provides a small reduction in D_95%_, even when the maximum target displacement is considerably larger than the margin size (ΔD_95%_ ≈ 0.97 when the maximum target displacement is 15 mm outside the PTV). SI and 3D motion caused much larger reductions in D_95%_. The most likely explanation for this effect is due to directional differences in dose gradients for co-planar treatment plans, with a relatively steep dose fall-off outside the target volume in the SI direction compared to AP. Movement of the target through these steep dose gradients with SI motion would likely cause a greater dose blurring and therefore a larger reduction in D_95%_. Another explanation for the motion-direction dependence occurrence can be realized when considering the cumulative effect of multiple gantry angles on the interplay and dose blurring effects. With zero couch rotation, SI motion acts perpendicularly to the beam and in the plane of the MLCs; this increases the contribution of the interplay effect. Depending on the gantry angle, AP motion could potentially be moving parallel to the beam and perpendicular to the MLC plane, eliminating the interplay effect and reducing intra-fraction motion effects. In this scenario, the only effect of motion would be an inverse square correction (which is accounted for in the algorithm), a small effect compared to a physical displacement of the target perpendicular to the beam. With non-coplanar beam configurations, these results will clearly be different.

Figure 3 demonstrates that the effect of motion on target dosimetry is dependent on the maximum sinusoidal target displacement, independent of the type of sinusoidal motion leading to this maximum (e.g. offset, amplitude or drift). In other words, Equation 3, and the corresponding data shown in Figure 3, hold true regardless of whether the motion tracks creating the displacement were generated with a drift, offset or variable amplitude. Data from the independent variable studies are indistinguishable from each other when plotted in the format shown in Figure 3d.

The cumulative effect of motion over several fractions for several different starting phases (Figure 5a) or motion tracks (Figures 5b and 5c) demonstrated that, while the cumulative D_95%_ was approximately equal to the average D_95%_ of the individual fractions, the cumulative D_05%_ was typically less than that of the individual fractions. The systematic peripheral cooling effect per fraction is present for most motion tracks so the cumulative effect of multiple fractions results in an average under-dosing in these regions. Conversely however, the more random, smaller hot spots near the center of the target become less prominent with increasing fractionation. Other combinations of motion tracks and fractionation schemes were calculated (data not shown), displaying similar effects to those shown in Figure 5. The data shown in Figure 5 is representative of the observed effects of multiple treatment fractions, but a more thorough analysis looking into the complex relationships between margins, drifts and fractionation schemes is beyond the scope of this current study.

## Conclusions

Motion-encoded dose distributions were calculated for multiple sinusoidal motion tracks applied to SNS IMRT plans for nine lung tumor patients. Further study needs to be done using actual tumor motion tracks as they become available. However, the results using these simulated data provide valuable insight regarding the relationship between treatment dynamics and tumor motion for SNS treatments, and also about the protective value of internal margins. As expected, the addition of an internal margin around the target forming the PTV reduced the potentially detrimental dosimetric impact of motion. For SI motion the margin can be reduced by an additional 5 mm while maintaining an acceptable dosimetry in the target (a change in D_95%_ of less than 3%), allowing for increased normal tissue sparing. This reduction can be increased even further for AP motion where SNS IMRT motion sensitivity appears to be less significant, with a co-planar beam arrangement. Even in the presence of moving MLC leafs, data from this investigation suggest that with careful selection of an internal margin, the dosimetric effects of motion can be successfully managed and the desired dose can be delivered to the target. Additionally, it was found that clinical target volume under-coverage due to motion is neither reduced nor truly propagates as treatment fractionation is increased.

## Consent

This study was reviewed and approved by Orlando Health’s institutional review board. It was determined by the IRB that no consent was necessary, as this study was a retrospective review of nine lung cases.

## Competing interests

The authors declare no conflicts of interests related to this investigation.

## Authors’ contributions

Each author has participated sufficiently in the work to take public responsibility for appropriate portions of the content. SLM, KML, BJW designed the study. SLM, BJW, APS performed the study and analysis. JMR, APS provided clinical assistance with patient planning. The manuscript was written by BJW; all other authors helped and approved the final manuscript.
